# Fragile X-Associated Neuropsychiatric Disorders (FXAND)

**DOI:** 10.3389/fpsyt.2018.00564

**Published:** 2018-11-13

**Authors:** Randi J. Hagerman, Dragana Protic, Akash Rajaratnam, Maria J. Salcedo-Arellano, Elber Yuksel Aydin, Andrea Schneider

**Affiliations:** ^1^Medical Investigation of Neurodevelopmental Disorders (MIND) Institute, University of California, Davis, Sacramento, CA, United States; ^2^Department of Pediatrics, University of California Davis School of Medicine, Sacramento, CA, United States; ^3^Department of Pharmacology, Clinical Pharmacology and Toxicology, School of Medicine, University of Belgrade, Belgrade, Serbia; ^4^Case Western Reserve University School of Medicine, Cleveland, OH, United States; ^5^Istanbul Faculty of Medicine, Istanbul University, Istanbul, Turkey

**Keywords:** fragile X-associated neuropsychiatric disorders, FXAND, *FMR1* premutation, FXTAS, FXPOI

## Abstract

Fragile X syndrome (FXS) is caused by the full mutation (>200 CGG repeats) in the Fragile X Mental Retardation 1 (*FMR1*) gene. It is the most common inherited cause of intellectual disability (ID) and autism. This review focuses on neuropsychiatric disorders frequently experienced by premutation carriers with 55 to 200 CGG repeats and the pathophysiology involves elevated *FMR1* mRNA levels, which is different from the absence or deficiency of fragile X mental retardation protein (FMRP) seen in FXS. Neuropsychiatric disorders are the most common problems associated with the premutation, and they affect approximately 50% of individuals with 55 to 200 CGG repeats in the *FMR1* gene. Neuropsychiatric disorders in children with the premutation include anxiety, ADHD, social deficits, or autism spectrum disorders (ASD). In adults with the premutation, anxiety and depression are the most common problems, although obsessive compulsive disorder, ADHD, and substance abuse are also common. These problems are often exacerbated by chronic fatigue, chronic pain, fibromyalgia, autoimmune disorders and sleep problems, which are also associated with the premutation. Here we review the clinical studies, neuropathology and molecular underpinnings of RNA toxicity associated with the premutation. We also propose the name Fragile X-associated Neuropsychiatric Disorders (FXAND) in an effort to promote research and the use of fragile X DNA testing to enhance recognition and treatment for these disorders.

## Introduction

Mutations in the *FMR1* gene are relatively common in the general population and create a spectrum of disorders, ranging from neurodevelopmental problems in childhood to neurodegenerative problems in aging. Two types of mutations are recognized, and each has a different pathophysiological mechanism leading to their corresponding phenotypes. The full mutation, which has >200 CGG repeats in the 5' untranslated region of *FMR1*, typically causes methylation leading to silencing of *FMR1* such that little or no *FMR1* mRNA and FMRP are produced. This leads to FXS, which is characterized by ID in 85% of males and 25% of females (1). The second type of mutation is the premutation, which ranges from 55 to 200 CGG repeats; individuals with the premutation are also called carriers. The pathophysiology of carrier involvement is caused by elevated levels of the *FMR1* mRNA leading to RNA toxicity, as described below. However, repeats in the upper end of the premutation often lead to mildly deficient FMRP levels as well, because translation of mRNA with >120 repeats is inefficient (2–4).

Only two disorders among premutation carriers have been recognized and named: the fragile X- associated Primary Ovarian Insufficiency (FXPOI) is characterized by menopause before age 40 and occurs in approximately 16–20% of female carriers, while the fragile X-associated Tremor/ Ataxia Syndrome (FXTAS) occurs in approximately 40% of older male carriers and 16% of older female carriers (5, 6). FXTAS and FXPOI are commonly recognized, but the most common problems of premutation carriers are psychiatric. However, these psychiatric problems are not typically recognized as related to the premutation because they do not have a fragile X- associated name. Therefore, this paper describes the fragile X-associated Neuropsychiatric Disorders (FXAND), bringing recognition to these problems by naming them.

## Premutation prevalence and molecular pathology

The premutation is common in the general population, occurring in approximately 1 in 200 women and 1 in 400 men (7). *FMR1*-mRNA levels are increased in individuals in the premutation range, with higher CGG repeat numbers correlating with higher mRNA levels (8). The high level of mRNA causes toxicity related to the sequestration of proteins that are important for neuronal function (9). Premutation neurons die more readily in cell culture (10), and they are more vulnerable to toxins in the environment, such as alcohol and pesticides (11). In addition, intracellular calcium dysregulation (12), oxidative stress, mitochondrial dysfunction (13, 14), chronic DNA damage repair changes (9) and the formation of the toxic protein FMRpolyG (15) are all related to the toxicity of the premutation. Ultimately, this toxicity can lead to the neurodegenerative disorder FXTAS.

FXTAS typically begins in the 60s and is characterized by the onset of an intention tremor followed by ataxia, which leads to frequent falling (11, 16). The MRI demonstrates global atrophy and white matter disease, usually in the middle cerebellar peduncles, periventricular area, splenium of the corpus callosum and insula (11, 17). Neuropathological studies have identified inclusions in neurons and astrocytes of those who have died of FXTAS (18). FXTAS inclusions can occur throughout the body, which helps to explain the fact that many carriers have symptoms involving a variety of organs, including irritable bowel syndrome and cardiac arrhythmias; moreover, these problems can add to the psychiatric symptoms that many carriers experience (11, 19). Cognitive problems are also common, beginning with memory and executive function deficits and later followed by further decline and often dementia, which occurs in approximately 50% of males with FXTAS (20). Women are relatively protected from FXTAS, presumably because approximately half of their neurons and glial cells express the normal X chromosome rather than the X chromosome with the premutation, so there is less associated toxicity.

The inclusions in FXTAS involve sequestered proteins and neurofilaments which are important for neuronal and astrocyte function; therefore, the formation of inclusions may impair the viability of these cells (9–11). There is also mitochondrial dysfunction in those with FXTAS, even in carriers before onset of FXTAS (13, 14, 21). Neuronal calcium dysregulation also occurs, as intracellular calcium levels are high in premutation neurons (12). Chronic DNA damage repair and iron dysregulation and sequestration in the CNS are also observed (9, 22). In addition, the formation of FMRpoly G, a toxic protein, has been documented in some patients with FXTAS as well as in premutation animal models (15); this protein forms because of repeat-associated non-AUG (RAN) translation of the prolonged CGG sequence in premutation mRNA (23). Moreover, these neuropathological mechanisms may take place not only in those with FXTAS, but perhaps in those with other premutation disorders such as FXAND and FXPOI as well.

FXAND refers to the neuropsychiatric problems that typically occur at an earlier age than FXTAS, and examples of these problems are described below.

## Premutation involvement throughout the lifespan

Recent studies of MRI results in premutation carriers over the lifespan have documented structural changes that can begin in childhood (24); indeed, we often see clinical involvement including visual spatial deficits in carriers who are infants, although they are far more subtle than what is seen in the full mutation (25). Wheeler et al. (26) found that premutation babies identified by newborn screening demonstrated a greater sensitivity to sensory stimuli compared to controls; furthermore, those at the upper end of the premutation demonstrated early delays, which is likely related to lowered FMRP levels. Farzin et al. (27) demonstrated a high rate of attention-deficit/hyperactivity disorder (ADHD) and autism spectrum disorder (ASD) in premutation boys who presented clinically. Carrier boys who were identified by cascade testing in a family also had similar problems, but at a lower prevalence compared to boys without the premutation. Clifford et al. (28) also found a high rate of ASD (14% in boys and 5% in girls) when assessing children with the premutation. Chonchaiya et al. (29) demonstrated that carriers who had seizures had higher rates of ASD and developmental problems than those without seizures. Therefore, pediatricians need to be alert for the premutation causes of neurodevelopmental and neuropsychiatric disorders and test for this mutation (26).

Because of the intrinsic vulnerability of premutation carriers, the usual detrimental effects of environmental, epigenetic or genetic factors may lead to more significant problems than in the general population (30). We have also found that 20% of carriers who present with ASD or ID have a second genetic hit (31). Thus, microarray studies or whole exome sequencing may reveal changes that can be additive to the premutation or explain more severe involvement.

## Neuropsychiatric problems

The extensive molecular pathology and mitochondrial studies that have been carried out in premutation carriers has led to a wealth of molecular information to link to the psychiatric problems that carriers experience (11, 12, 32, 33). We know that psychiatric problems (usually depression and/or anxiety) occur before the neurological problems develop in those with FXTAS (34, 35). The molecular pathology, including calcium dysregulation, mitochondrial pathology, oxidative stress, chronic DNA damage repair, and inclusion formation, occurs in the neurons and astrocytes throughout the brain, including the amygdala (9, 12, 32, 36).

## Anxiety

Anxiety is the most common problem that carriers experience and typically begins in childhood (37). Cordeiro et al. (37) studied 35 premutation carriers between ages 5 to 23 (mean 11.3; *SD* 4.3) with the standardized Anxiety Disorders Interview Schedule for DSM-IV (ADIS-IV) and found that 70.6% met criteria for at least one anxiety disorder, compared to 22.6% of controls and 9.8% of the general population in this age range. Amongst the 35 carriers, the anxiety disorders most frequently diagnosed were Generalized Anxiety Disorder, Specific Phobia, Social Phobia or Obsessive-Compulsive Disorder. Schneider et al. showed an elevated rate of self-reported obsessive-compulsive symptoms in female premutation carriers compared to control females (31). Previously, this was thought to be related to raising a child with the full mutation FXS; however, in the reported study, none of the females had affected children.

Sometimes anxiety may be related to the sensitivity that carriers experience with environmental stimuli, something that was noted in babies with the premutation (26). Many carriers tell their clinician that eye contact makes them uncomfortable or anxious, so they either avoid eye contact or learn to force themselves to make eye contact as they grow older. This is a milder version of what is seen in those with FXS, where a severe deficit in GABA inhibition occurs along with a severe deficit of habituation to all sensory stimuli (38). One study documented a mild GABA deficit in premutation carriers with EEG/ ERP studies (39), which may exacerbate the anxiety symptoms to sensory stimuli.

Enhanced glutamate activity in premutation neurons leading to the Ca^+2^ dysregulation has been documented by Cao et al. (40, 41). In addition, in postmortem brains of those with FXTAS, Pretto et al. (42) demonstrated decreased cerebellar expression of the astrocytic glutamate transporter EAAT1, as well as decreased expression of mGluR5 in 16 brains compared to controls (42). They suggested that decreased uptake of the excitotoxic neurotransmitter glutamate may add to premutation toxicity; additionally, decreased expression of the mGluR5 receptor may be secondary to the over-activity of this pathway in view of the lowered levels of FMRP found in the cerebellum of these carriers (42). Hessl et al. (2, 43) have also demonstrated underactivity of the amygdala in adult males with the premutation. This correlated not only with mRNA levels, but also with a mild deficit of FMRP levels. The decreased amygdala activation in this group of patients was significantly linked with self-report of psychological symptoms on the Symptom Checklist-90-Revised (43).

## Depression

Depression has been commonly described in both male and female premutation carriers, and rates of depression are higher amongst carriers than controls or the general population (34, 44–46). In controlled studies, depression occurs in approximately 40% of premutation carriers, while patients with FXTAS were found to have a 65% lifetime prevalence of mood disorders (45). In the context of FXTAS, depressive symptoms are typically described before the onset of motor symptoms, which suggests that depressive symptoms could present prodrome of later motor impairments in patients who develop FXTAS (47).

One of the first studies of depression in carriers evaluated 85 women and found a significant positive relationship between repeat size and depression (48) such that those with repeats above 100 scored significantly higher on the Depression subscale of the Symptom Checklist-90-Revised (49). Similar conclusions were obtained in a study which included 119 males and 446 females aged 18–50 (50). Roberts et al. (46) and others have published that the relationship between CGG repeats and the prevalence of major depressive disorder is curvilinear, such that the middle range of 70–100 repeats confers the greatest risk, while repeats on the lower-end and higher-end of the premutation range confer lower risks of psychiatric problems. However, the *onset* of depression in carriers is not associated with number of CGG repeats (47). The same curvilinear association seen with psychiatric problems has also been seen in the risk for FXPOI (51), as well as the risk for a variety of other health problems such as fibromyalgia, chronic fatigue and chronic pain (52).

Other genetic factors, such as allelic variants in background genes, may impact the incidence of depression or anxiety as well. Hunter et al. (53) evaluated the effect of single nucleotide polymorphisms (SNPs) of the corticotropin releasing hormone receptor 1 locus (CRHR1), which controls the hypothalamic-pituitary axis (HPA) axis and the response to stress, particularly the stress of raising a child with FXS. Although they did not find a correlation with depression, they did find 2 SNPs of CRHR1 that significantly correlated with social phobia in mothers of children with FXS; however, this correlation was not found in premutation carriers without children with FXS. Multiple studies have demonstrated significant stress associated with raising a child with FXS (54–56), particularly if the child with FXS has significant aggression toward the carrier mother (57). Seltzer et al. (56) found that women with the mid-range of CGG repeats (85–110) had the highest depressive symptoms compared to carriers with low or higher repeat numbers when they had a high number of negative life events.

The median onset age of major depressive disorder in individuals with the *FMR1* premutation are significantly higher than in the general population (47). In the context of neurodegenerative changes, carriers become more sensitive to stress factors later in life, which may explain this later age of onset (47). The brain volume in carriers is reduced significantly more over time when compared to controls (24). This may reflect accumulative RNA toxicity and could impact the limbic system and induce depressive symptoms with aging, something that is common amongst other neurodegenerative disorders (58).

Regarding gender differences, females experience an earlier onset of depression symptoms than males (46, 47). One of the most important causes of earlier onset in females could be intense stress of parenting children with FXS, as discussed above (47). However, depression also occurs in women before having children with FXS (59). Kraan et al. studied a unique cohort of 24 female carriers (mean age was 30.5 years) without affected children and demonstrated that these individuals reported significantly elevated symptoms of depression relative to controls. Thus, the premutation itself enhances the risk for mood disorders independent of the stress related to raising children with FXS. Therefore, screening for depression and other psychiatric disorders in premutation carriers, before they become parents, is recommended (60). In another study of 83 premutation women, lower optimism and lower religious participation were linked with lifetime history of major depressive disorders (61).

The elevated prevalence of depression in individuals with the *FMR1* premutation is a clear feature of the premutation. As described above, there is complex set of factors that could contribute to the occurrence of depression in premutation carriers beyond RNA toxicity, including environmental, background genetic factors and likely epigenetic factors, all of which require further study. An important clinical issue is the treatment of depression once it is recognized. The use of antidepressant medication, including selective serotonin reuptake inhibitors (SSRIs) can be utilized to the benefit of the patient and should be considered by the clinicians who recognize these problems and treat carriers (62, 63).

## ADHD and linkage to substance abuse

Farzin et al. (27) found an increased prevalence of ADHD in proband boys with the premutation who were seen in clinic compared to brothers without the premutation and non-proband carriers. ADHD was seen in 93% of probands, 38% of non probands, and 13% of controls. Bailey et al. (64) conducted a survey to assess co-occurring conditions in children with full mutation and premutation and found that 45% of males and 14% of females with the premutation were diagnosed or treated for attention problems; hyperactivity was also reported in 30% of male carriers.

Earlier studies in adults found increased frequency of ADHD as reported by daughters of carrier fathers (65). The familial aggregation of the disorder was later analyzed in females aged 18–50 by Hunter et al. (53). They reported a non-linear effect with respect to CGG repeat size on ADHD related symptoms using an adult ADHD rating scale with significantly higher scores in carriers compared to controls.

Regarding substance use, two studies have reported that excessive alcohol consumption and drug use is relatively common in carriers compared to controls (65, 66). In addition, multiple case studies have documented numerous individuals who have abused substances including alcohol, cocaine, and marijuana (67–69). The high rates of ADHD, anxiety, and depression found amongst carriers provides a possible explanation (self-medicating) for why illicit drug use and/or excessive alcohol consumption are common in carriers. In addition chronic pain related to neuropathy, fibromyalgia or migraine headaches, which are common in carriers (70, 71) can also encourage self-medication and contribute to the use of drugs, including opiates and excessive alcohol (62).

These problems related to substance abuse have significant consequences for the brain. Excessive alcohol consumption decreases white matter integrity, disrupts myelination, and induces neuroinflammation (72–74). Moreover, illicit drugs such as methamphetamine and cocaine can lead to neuronal oxidative stress (75–77), which is already present in aging carriers and can further decrease the survival of neurons. Thus, abuse of any of these substances may increase the likelihood of developing FXTAS, and rapidly increase the progression of FXTAS symptoms (62, 67, 69, 78).

## Chronic pain & fibromyalgia

The association of muscle pain and fibromyalgia with premutation carriers was first described by Coffey et al. (70). They reported a significantly higher prevalence of chronic muscle pain, defined as persistent myalgia for more than 2 months unrelated to injury, in female premutation carriers both with and without FXTAS and a significant increase in fibromyalgia in the FXTAS group (43.8%) compared to controls (9.4%). Additional reports have shown similar findings (6, 79). Leehey et al. (79) described the clinical presentation on a series of cases, all of them reported early onset of localized chronic muscle pain, before age 50, and progression to fibromyalgia later on. Rodriguez-Revenga et al. found a penetrance of ~25% (*n* = 90) of chronic muscle pain among females with the *FMR1* premutation; their findings were statistically significant compared with the prevalence of 2% in individuals over 50 years among the general population (6).

The appropriate management of chronic pain and fibromyalgia in premutation carriers is challenging since the long-term use of opioids has been associated with changes in the white matter (80–82) and cell death (83). Chronic use of opioids as well as opioid overdoses have been described to trigger the progression of white matter disease and accelerate the neurological decline in FXTAS (69, 78, 84).

## Chronic fatigue

Chronic fatigue is a common symptom of premutation carriers with and without FXTAS and it has a significant effect on their daily lives (85, 86). It is likely associated with the mitochondrial dysfunction described previously in premutation carriers; previous studies have linked chronic fatigue in carriers with the severity of mitochondrial dysfunction (32, 33).

There is a high prevalence of sleep apnea in patients with FXTAS, which can be also be associated with chronic fatigue (87). Increased BMI is also thought to be indirectly related to fatigue because of its relation to sleep apnea, diabetes and coronary artery disease. Summers et al. (85) reported that premutation carriers with FXTAS are more affected by fatigue than the individuals with premutation without FXTAS and the control group. Premutation carriers without FXTAS show an intermediate level between FXTAS patients and controls. They mentioned that depression is correlated with fatigue and that treatment of depression reduces the fatigue scores of patients (85, 88).

## Sleep disturbances

Sleep problems commonly occur in individuals with depression and anxiety; however, in those with the premutation, sleep problems are usually seen even before the onset of neuropsychiatric problems. Sleep problems were the most common finding amongst adult carrier daughters of men with FXTAS, and the incidence of sleep problems among these women was significantly increased compared to controls (89) and this may also be related to sleep apnea (87). Furthermore, opioid use, which is associated with premutation carriers as discussed previously, can also increase the risk of sleep apnea (90). Bailey et al. (64) also found an increase in sleep problems in younger individuals with the premutation compared to controls; moreover, several commonly co-occurring conditions, such as ADHD and anxiety, were found to increase the prevalence of sleep disturbances seen in individuals with premutation (64). These sleep problems are likely related to the documented GABA deficit associated with carriers (39), as the GABA system has such a significant role in sleep (91)

## Autoimmune problems

Although autoimmune problems are not neuropsychiatric problems, they are also associated with the *FMR1* premutation and may exacerbate neuropsychiatric problems (70, 92–94). Interestingly, autoimmune diseases occur predominantly in women who are carriers, while males with premutation rarely experience autoimmune problems. Winarni et al. (93) studied 344 carrier women ages 18 to 91 and found that 45% of carriers had at least one immune-mediated disorder (IMD), compared to 28% of 72 controls. Among carriers, autoimmune thyroid disorder was the most common (24.4%), followed by fibromyalgia (10.2%), irritable bowel syndrome (IBS; 9.9%), Raynaud's phenomenon (7.6%), rheumatoid arthritis (RA; 3.8%), Sjogren syndrome (2.6%), systemic lupus erythematosus (2.03%), and multiple sclerosis (1.74%). However, only autoimmune thyroid disorder and fibromyalgia were significantly increased in carriers compared to controls. Of 55 carriers age 40 or older with FXTAS, 72.73% had at least one IMD, compared to 46.54% of those without FXTAS and 31.58% of controls. The estimated odds ratio (OR) for IMD is 2.6 (*p* = 0.015) for women with FXTAS relative to those without FXTAS. The likelihood of IMD in carriers with or without FXTAS was also significantly higher than for controls (OR 2.1, *P* = 0.034; OR 5.5, *P* < 0.001, respectively). Jalnapurkar et al. hypothesized that autoimmune problems could exacerbate emotional problems and accelerate the onset of FXTAS (94). The pathophysiology of autoimmune disorders could also lead to emotional problems through mechanisms of inflammation, immune dysregulation, stress, or miRNA dysregulation (95–97).

## Treatment of FXAND

Treatment of depression and anxiety disorders usually involve selective serotonin reuptake inhibitors (SSRIs) or serotonin and norepinephrine reuptake inhibitors (SNRIs) and these medications are typically helpful for the psychiatric symptoms in FXAND (62, 63, 88); however, controlled studies have not been carried out specifically in those with the premutation. The importance of identifying the premutation as the etiology of FXAND is to treat the oxidative stress, mitochondrial dysfunction, and other complicating comorbidities such as hypertension, migraine headaches, thyroid dysfunction, and chronic pain that are associated with the premutation. It is also important so that providers can recommend the avoidance of toxins in the environment such as excessive use of alcohol or opioids (69, 78, 98), which can cause more CNS disease; exposure to pesticides, which can worsen white matter disease and brain atrophy (99); isofluorane use in aging patients, because this may be the most toxic anesthetic agent and it may precipitate FXTAS symptoms (100). In general, we recommend daily exercise to help with depression or anxiety and also stimulate neurogenesis and improve mitochondrial function (62). In addition, sleep disturbances, chronic pain symptoms, and anxiety, which are all common problems in those with FXAND, are likely to improve with the use of cannabidiol (CBD) due to GABA enhancement; however, this recommendation requires further controlled trials. Though CBD may be helpful, the avoidance of tetrahydrocannabinol (THC) is recommended because of the high risk of psychotic thinking associated with its use, and this may be particularly important in carriers. Lastly, the development of new and more powerful antioxidants that also stimulate mitochondrial biogenesis are likely to be beneficial for carriers; these include idebenone and Anavex 2-73, and controlled trials for these treatments are needed (101, 102).

## Conclusion

The recognition of FXAND is important because it identifies a large group of premutation disorders beyond FXPOI and FXTAS that still cause significant morbidity to numerous patients with the premutation. Most of these individuals do not meet the established criteria of FXTAS because they do not experience persistent tremor and/or ataxia, nor do they have the CNS findings of white matter disease in the classical location of the middle cerebellar peduncle found in FXTAS as defined in the literature (5, 11). The delineation and recognition of FXAND is important to guide further research regarding neuropathological mechanisms that lead to neuropsychiatric problems including mitochondrial dysfunction, RNA toxicity, and the production of FMRpolyG. The recognition of FXAND will also facilitate the involvement of professionals in behavioral sciences to target the treatments that will be helpful for this group of neuropsychiatric disorders.

## Author contributions

All of the authors participated in drafting the manuscript. RH additionally revised it critically for important intellectual content. All of the authors gave final approval of the version to be submitted.

### Conflict of interest statement

RH has received funding from Roche, Novartis, Neuren, Marinus and Alcobra for carrying out treatment studies in patients with fragile X syndrome. She has also consulted with Fulcrum, Ovid and Zynerba regarding treatment studies in individuals with fragile X syndrome. The remaining authors declare that the research was conducted in the absence of any commercial or financial relationships that could be construed as a potential conflict of interest.
